# Application of the Computed Tomography Method for the Evaluation of Porosity of Autoclaved Materials

**DOI:** 10.3390/ma15238472

**Published:** 2022-11-28

**Authors:** Anna Stepien, Małgorzata Durlej, Karol Skowera

**Affiliations:** Faculty of Civil Engineering and Architecture, Kielce University of Technology, al.1000-lecia PP 7, 25-314 Kielce, Poland

**Keywords:** autoclaved, CT analysis, silica, bricks, sand

## Abstract

This article describes the use of recycled glass sand in the production of autoclaved products. Traditional autoclaved bricks consist of crystalline sand, lime and water. The conducted research aimed at the complete elimination of quartz sand in favor of glass sand. This work focuses on porosity as the functional property of the materials. The aim of this article is to determine the number and structure of the pores of autoclaved bricks. Two types of research were carried out: (a) non-destructive, i.e., computed tomography examination as a pictorial and quantitative method and (b) mercury porosimetry as a quantitative method, i.e., a test that exposes the porous skeleton of the material for destruction. The tests showed the presence of pores with a size in the range of 0.1 ÷ 100 μm, and the volume of voids in the material was determined at the level of about 20% for the sample modified with glass sand (GS) and for the reference sample made of traditional silicate brick. In order to complete the research on the internal structure of autoclaved bricks, microstructure studies were performed using a scanning electron microscope (SEM). The tests showed the presence of tobermorite in the reference sample (with 90% QS-quartz sand) and the presence of natrolite and gyrolite in the sample modified by glass sand (90% GS).

## 1. Introduction

This article deals with the topic of sustainable construction in the context of ecology and material deficits. This work is a continuation of considerations on the possibility of using glass sand from glass recycling in materials resulting from hydrothermal treatment, i.e., subjected to conditions of increased pressure and temperature [1,2,3] (a technology which is popular largely in Western Europe and Central and Eastern Europe) in which the main substrate is quartz sand. In order to relieve the environment, alternatives to the use of quartz sand are being searched and tested. A wide spectrum of applications shows the share of glass sands in the modification of concrete and bricks. The use of aggregates in recycled components is conducive to a sustainable economy, and the appropriate selection of modifier may additionally reduce heat losses in a building and improve the economic situation of the investor (lower costs to heat the building), which contributes to the improvement of environmental conditions (lower energy consumption) and is now in line with in the issues of sustainable construction. The functional properties of construction materials include many factors, such as the quality of the substrates constituting the raw material mass of the materials, the production technology and the appropriate operation, although the latter depends on environmental factors. The functional properties, on the other hand, reflect the structure of the materials, i.e., porosity or appropriate bonds and phase transitions. The sand-lime products referred to in this article belong to the group of porous materials [3]. In nature, porous substances occur naturally, including various building materials, including concrete and bricks. The porosity of the materials, and in particular the materials that make up the structural external partitions in buildings, is one of the main factors determining the current modifications, and it is favored by the trend related to sustainable development and construction, as well as current climate change. The pore size plays a major role in determining, for example, the mechanical, thermal insulation and acoustic properties of a material as well as its permeability [4]. The porosity of a material depends on the size of a solid which is determined by the size and number of voids inside the material and hence dictates their performance properties, ranging from strength to density, thermal insulation or sound insulation [5]. This feature of the materials depends on their composition and production technology and often reaches up to 75% of the total volume of voids in the material (cellular concrete) [4,6,7]. This phenomenon may be beneficial in the case of low-rise buildings made of materials with good thermal insulation, but it does not work when the factors determining the construction are, for example, good density, strength or acoustic insulation. The determinant of all these dependencies in the material is porosity. Research methods make it possible to determine the number of open and closed pores [8]; however, depending on the research method and the selected apparatus, the results may be different. This article presents two methods of determining the porosity of a material: CT analysis and mercury porosimetry. These studies are important because solutions favoring the utilization of recycled materials or aggregates (e.g., silicate aggregate, glass cullet) are sought in modern construction, including fly ash and amorphous glass powder replacements [9,10,11,12,13]. In addition, a very important indicator in the research is the quantity, form and process of crystallization of the C-S-H phase. In hydrated cements, roughly 60–80% of the volume of the formed solid comprises the hydrated calcium silicate phases abbreviated as C-S-H. Many studies have been conducted to develop an understanding of the structure and properties of the C-S-H phase in building materials [1,14,15,16,17,18]. Due to its manufacturing technology, the internal structure of autoclaved materials, especially silicate bricks, is radically different from that of concrete products. The structure of concretes comprises the amorphous C-S-H phase (calcium silicate hydrates). Materials formed in the vicinity of 70 °C have in their structure the C-S-H phase, which has the ability to crystallize at elevated temperatures as tobermorite (C-S-H I) [19]. Currently, radiation is often used to observe the microstructure of any material in various industries, sectors or research fields [20]. Civil engineering is one of the main fields of engineering in the context of the application of various materials in the construction industry [21]. Due to the shrinking resources of natural aggregates and the so-called overproduction (high material consumption, including the use of natural aggregates in a very short time) and rising inflation, researchers and entrepreneurs attempt to limit the ineffective use of natural resources and hence reduce funding. CT analysis seems to be a viable test, because it is non-destructive, and the material sample itself can be examined at various intervals by observing the changes that have occurred over the course of material’s usage. 

CT analysis has already found a wide variety of applications in the non-destructive testing of concrete structures [20,21]. The difference between autoclaved bricks and concretes is two-fold:

(1) The production process of silicate bricks takes a maximum of 8 h, after which we obtain a brick with a strength of 15–20 MPa, from which buildings of up to five floors can be made. This type of bricks is a natural building material intended for the construction and partition walls with a heat transfer coefficient (U) in the range of 0.46–0.72 W (m^2^·K) [22], which is related to their high density and good sound insulation (1600–2000 kg/m^3^) [23]. Such time is obtained due to the use of autoclaves and hydrothermal conditions (elevated pressure + temperature of 203 °C). Cellular concrete is produced in a similar way, but the temperature limit is set to 100 °C;
(2) The difference in the microstructure of the materials produced, i.e., the structure of the concrete, by about 70%, consists of the amorphous C-S-H phase, capable of crystallization, while the structure of silicate bricks consists of 90% (by mass) tobermorite crystalline phase (crystallization of the C-S-H phase into tobermorite at elevated temperatures in traditional silicate bricks) and/or gyrolite or natrolite (crystallization of the C-S-H phase toward natrolite or gyrolite in silicate bricks modified with glass sand associated with elevated the Na_2_O content). Thus, in terms of thermodynamic considerations, silicate bricks are more stable and resistant to weather conditions (e.g., fluctuating temperature, especially in the period of negative and high temperatures). The C-S-H phase is metastable and retains its properties up to temperatures of 25–30 °C. The analysis of the pore content in concrete is also important since it is believed that the formed pores can serve as an effective protection against the harmful effects of frost [24,25]. If the total air content in the concrete is between 4% and 7%, the average distance to the nearest air quantity values (spacing factor) L is below 0.20 mm or 0.22 mm, the specific surface area of the pore system is in the range of 16–24 mm^−1^ and the minimum air content in the pores is smaller than 0.3 mm (A300) by at least 1.5%. The basic method of examining the concrete’s structure is a qualitative image analysis of it, followed by computer image analysis, leading to quantitative results [26,27,28,29].

### Sustainable Construction in the Context of Ecology and Economy

**Figure 1:**
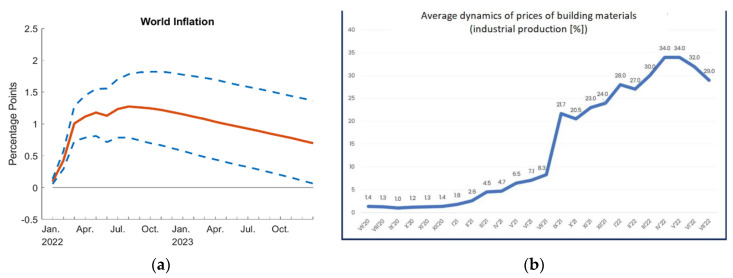
Inflation level and average growth rate of building materials prices. (**a**) Inflation in the world after 2019 [24]. (**b**) Increase in prices of building materials after 2019 [32].

The construction industry faces challenges such as progressive civilization, consumption and material changes, the geopolitical situation in the world and conflicts which cause changes in the natural environment of mankind. These changes often interfere with the landscape and the structure of the natural environment and contribute to the economic crisis (currently the deficit in coal and gas in Europe, or the growing inflation [30] in the world—the highest in 20–40 years depending on the country, as seen in Figure 1). The figure plots (Figure 1a,b) reflect the response over time of world GDP and world inflation, which is predicted to rise in the future [31].

The biggest problem of the construction industry is the excessive use of natural aggregates for the construction of new facilities, which is related to the so-called overproduction and excessive consumption of natural aggregates, including quartz sand, and the further heating of buildings with solid fuel along with the development of the refining industry, which leads to progressive climate change (global warming associated with an increase in CO_2_ levels in the atmosphere) [33,34,35,36,37]. In June 2012, the Rio + 20 Conference was held in Rio De Janeiro. This United Nations conference raised the topic of sustainable development again and was called ‘Rio + 20’ to commemorate the conference that took place at the same place 20 years earlier, also known as the Earth Summit 2012. Over 500 panels and accompanying events were devoted to two main issues:

(1) Green economy contributing to solving social problems in a sustainable manner, including, in particular, poverty eradication;
(2) Institutionalization of global cooperation for sustainable development, which is to lead to a greater harmonization and effectiveness of these activities [33]. Participants focused on seven priority areas for the international community, namely workplaces, energy, sustainable urban development, food security and sustainable agriculture, water, protection of the oceans and response to natural disasters. In the official international discourse, there were issues related to green economy, social reporting and the product life cycle [37], so that it could be eventually reused (recycling). An example of such a material is glass cullet or silicate aggregate (aggregate after demolition of buildings made of silicate bricks and used as a road foundation).

The declaration from the Earth Summit in Rio De Janeiro in 1992 already informed about the abuse and the resulting depletion of natural resources, but the rapid changes could not be brought under control. This was related to the years of occupation and wars that took place in the 20th century, after which countries, including Poland, had to rebuild and reorganize. Faced with unfavorable past experiences and challenges in the future, the construction industry is expected to change. Some of these changes can be stopped, others not. Therefore, the modification of materials today should be approached carefully, both from an ecological and economic point of view. An important factor of the 21st century is spreading awareness about recycling and fighting to improve environmental conditions to reduce energy losses related to, among others, heating buildings, hence reducing CO_2_ emissions to the atmosphere to fight the aforementioned material overproduction. The current requirements for the heat transfer coefficient U (m^2^·K) for materials are presented below. This coefficient depends on the porosity of the material [38,39,40]. Currently, every investor planning construction has to choose pro-ecological and pro-economic solutions and technologies [39]. Another important document that brings together technical requirements in the context of sustainable construction are the WT Standards [38,39] for buildings with a heat transfer coefficient (e.g., external wall: 0.2 W/(m^2^·K), roof: 0.15 W/(m^2^·K), floor on the ground: 0.3 W/(m^2^·K)) [39].

## 2. Materials and Research Methods

**Figure 2:**
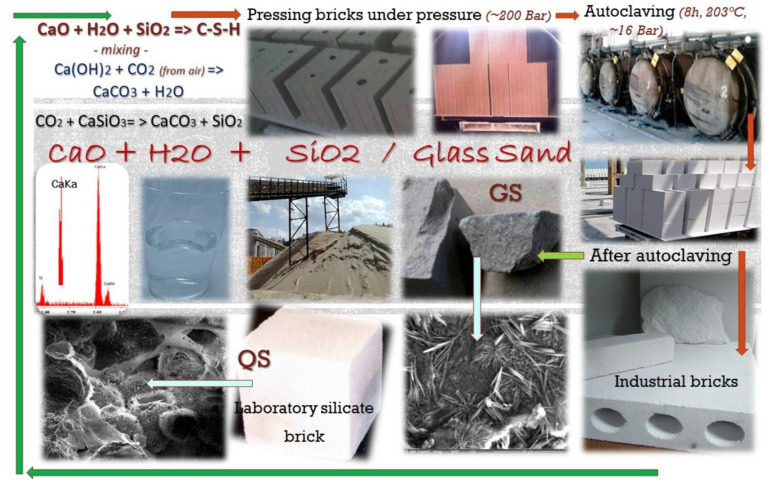
Production flow of traditional sand-lime bricks.

Two types of research were carried out on the internal structure of porous material: CT computer tomography and mercury porosimetry. The tests were carried out in accordance with the intended use of the described equipment (paragraphs above) and on the basis of the following standards: PN-EN 772-13: 2001, CEN. PN-EN 1996-2, CEN. PN-EN 771-2 and PN-EN 1936: 2010, PN-EN 993-1: 2019-01 [40,41,42,43,44]. The material that was analyzed are two types of bricks: (a) traditional and (b) modified brick with recycled glass sand. Glass sand from recycled bottled glasses was used for modification, with a grain size ranging between 80 and 160 μm. It was amorphous sand. The composition of traditional silicate materials is based on: sand, lime and water, i.e., natural substrates contents. Autoclaved materials are products created in the autoclaving process (Figure 2), i.e., under hydrothermal conditions, i.e., increased pressure and high temperature, and the process of their production usually ends within 8 h (1 h + 6 h + 1 h) [1]; the term autoclaving is related to the process taking place in hermetically closed autoclaves, heated with fuel (e.g., coal). In autoclaves, under conditions known as hydrothermal (hydro = water, thermal = heat), chemical processes and reactions that guarantee the appropriate strength and quality of the products produced in this way take place. The water temperature in the devices is stabilized or limited by the boiling point of water, which is 100 °C at a normal atmospheric pressure. Increasing the pressure in a closed autoclave makes it possible to achieve a relatively higher temperature (180–200 °C). The pressure in the autoclave is limited by an automatic pressure valve [45,46,47].

Tested materials consisted of a low binder content (7% lime by mass) and were abundant in silica up to 90% by mass modified with broken amorphous glass. Glass sand (GS) is characterized by a high sodium content. The presence of aluminum in the sample may affect the swelling of the material; therefore, an important aspect of the test is to perform the elemental composition analysis to acquire data on the amount of aluminum compounds in the tested material. The analyzed samples were manufactured in 2015 and the process of changes in the materials were observed. This process is important because glass sand is metastable (it crystallizes with increasing temperatures or pressure changes). It was shown that the crystallization proceeded toward natrolite and gyrolite, which is justified by the presence of sodium ions coming from the recycled glass. The sample was stored at a temperature of 23–26 °C for the entire period (for the period of 6 years until the next tests were conducted, i.e., April 2021). The compressive strength of laboratory samples for brick modified by glass sand (GS) was 20 MPa. The hydration temperature of the binder (CaO) with water (H_2_O) in the presence of sand (SiO_2_) was 86 °C for the reference mass (with QS) and 42 °C for fully modified GS bricks.

### 2.1. Computed Tomography

**Figure 3:**
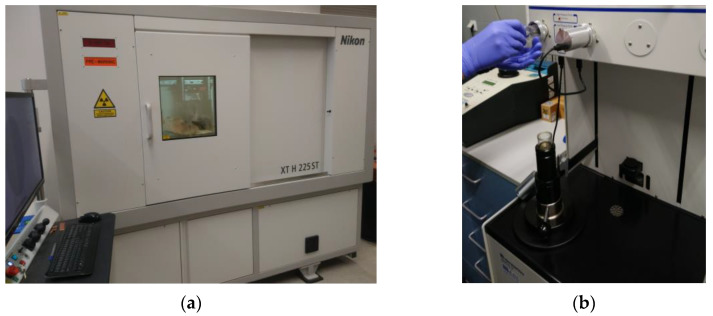
Basic devices used to measure the porosity of traditional silicate materials and those modified with glass sand: (**a**) Nikon XT H 225 ST computer tomograph; (**b**) QUANTACHROME ULTRAPYC 1200e mercury porosimeter.

In order to visualize the arrangement of pores in the studied materials, the following analyses were performed (Figure 3): micro-CT analysis (computer tomography), mercury intrusion porosimetry and SEM (scanning electron microscopy).

Micro-CT analysis was performed on a Nikon XT H 225 ST CT scanner (Minato-ku, Japan) (Figure 3a). The industrial CT scanner is designed for non-surface analyses and visualization studies that provide insight inside the examined material. The test is especially designed for specimens that are too large or heavy for other systems with a similar measurement range. The system can be equipped with three types of sources: reflective 225 kV, transmissive 180 kV and rotary 225 kV. Micro-CT analysis enabled measurements of the external and internal geometry of two types of autoclaved materials: (a) traditional bricks and (b) bricks with glass sand.

Porosity tests were performed using a QUANTACHROME ULTRAPYC 1200e helium pycnometer (Boynton Beach, FL, USA) on irregularly shaped samples weighing approximately 10–25 g (Figure 3b). The helium pycnometer used in this study is a fully automated piece of equipment.

Microstructure analysis was performed on a Quanta Feg FEI Company scanning electron microscope (with magnification of 14 to 1,000,000× and accelerating voltage of 200 V to 30 kV).

**Figure 4:**
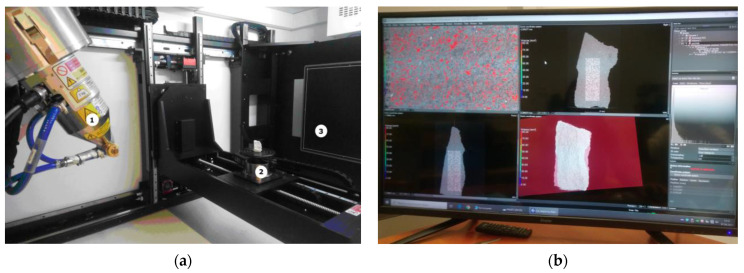
The method of performing the examination with the use of a computer tomograph. (**a**) Test method. Micro-CT analysis (1—reflection lamp, 2—rotary table with the sample, 3—detector). (**b**) Analysis of the sample in VG Studio Max.

The tomographic examination of samples of autoclaved materials included scanning the material (Figure 4a), digital image analysis and determination of the total air content in the analyzed samples. Using a spatial X-ray beam and a panel detector, after the object achieved a full 360° rotation, a series of photos of the object were obtained, which, after determining the center of rotation of the sample and pre-processing, were assembled into a 3D model (Figure 4b). The accuracy of the final projection depends on the number of projections made for a full revolution. Computed tomography allows one to analyze the internal structure of the samples without destroying them. Thanks to this method, a 3D image of the entire volume of the object or its selected fragments is obtained [26]. In the case of the conducted research, part of the material was separated in relation to the sample, which made it possible to carry out the analysis in selected sections of the sample and at different depths. The test consisted of directing the X-ray beam at the tested object and recording its intensity on the other side on the detector. X-ray radiation, like radiation from other parts of the electromagnetic spectrum, can be absorbed and scattered by matter [10]. Creating a tomographic image is based on measuring the absorption of radiation passing through an object. The volume of the object is divided into small cells (voxels in 3D technology, the equivalent of a pixel in 2D technology), in which the linear radiation absorption coefficient is the same. The distribution of the radiation absorption coefficients is calculated by a computer [26].

### 2.2. Mercury Porosimetry

**Figure 5:**
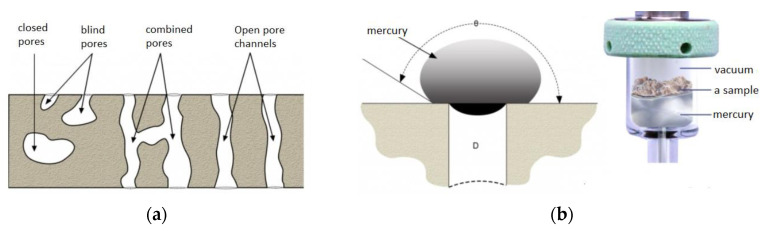
Example of mercury penetration into material; (**a**) a schematic illustration of the types of pores; (**b**) contact of mercury drop with porous material; illustration of mercury saturating a sample in a penetrometer [48].

Mercury intrusion porosimetry (MIP) is one of the basic research methods used for determination of pore structure, size and pore distribution. This method is based on the experimental determination of the so-called capillary potential curve (capillary pores) linking the volume of mercury pressed into a porous material sample with the pressing pressure (Figure 5). The standard model of the shape or architecture of pores assumed in mercury intrusion porosimetry is the capillary model, in which the pore space is formed by a bundle of capillaries with a stochastic distribution of diameters penetrating the entire sample of porous material [48,49]. As a non-wettable liquid, mercury does not spontaneously penetrate the pores of the tested material; therefore, its intrusion into the pores of the sample requires the use of appropriate pressure. As the set pressure increases, mercury penetrates smaller and smaller pores. On the basis of the measured values of pressure and mercury intruding into the sample, the measuring apparatus determines the volume of the pores and their distribution [50,51,52].

## 3. Results

The tests were used to determine the distribution and number of pores in the tested materials as well as their pore geometry. Simultaneously with the mercury intrusion porosimetry and Micro-CT analysis, the material was analyzed using a scanning electron microscope. Photos from the measurements of the samples are presented on the plane and in space as images of the distribution and size of air pores in the analyzed area of the tested material.

### 3.1. CT Tomography and SEM

**Figure 6:**
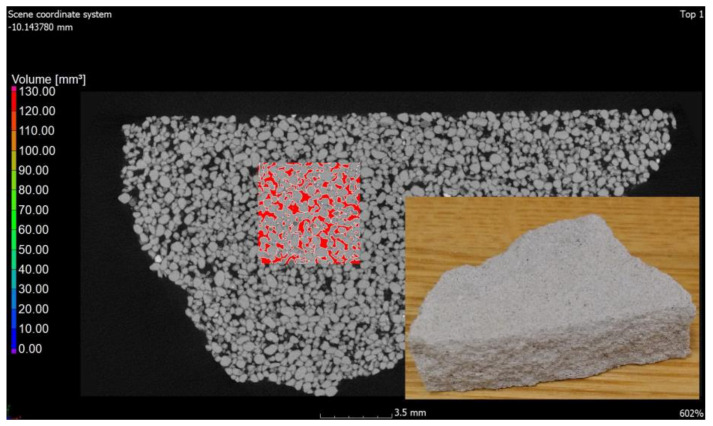
Photo of traditional material (reference sample) examined with computed tomography.

**Figure 7:**
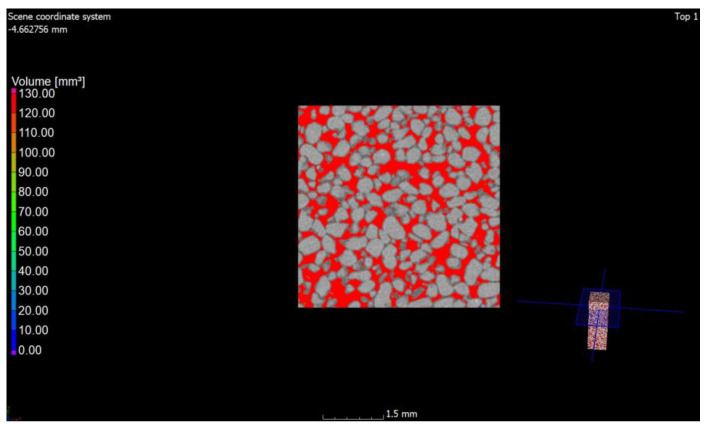
Cross-section of a sample in traditional brick. Red spots are visible free spaces that were formed at the interface between SiO_2_ and CaO grains and after the hydration and autoclaving process.

Computed tomography was used as a form of imaging the arrangement and shape of the pores, and then a study was carried out to determine the porosity of the materials. Under the influence of microstructural changes (crystallization of the amorphous phase), the porosity changes. The CT scan was performed with a reflection lamp (reflection target). The maximum capacity of this lamp is 225 kV and 225 W. The scan was made without a filter at 180 kV and 139 uA current. The exposure time was 250 ms. A total of 4476 projections were made and assembled into a 3D model. Each projection was generated with four frames per projection. The size of the voxel, i.e., the resolution, was 15 μm. All the parameters were selected experimentally to ensure the best image quality. During the CT scan, the pores were determined using the porosity/inclusions analysis tool in VG Studio Max. In order to separate the material from the voids, the value of the so-called voxel gray threshold was defined (the voids corresponded to the shades of black and very dark gray, and the remaining areas were the material). All voxels corresponding to the darker areas were identified and counted, thus obtaining the percentage and volume pore content of the entire sample volume as well as the volume of each individual pore. Pores with diameters smaller than the obtained resolution cannot be detected. The pores were sorted by volume and presented on a 3D model in a self-selected color scale. Blue-color pores correspond to pores in smaller volumes, while red ones correspond to higher volumes (Figure 6 and Figure 7).

**Figure 8:**
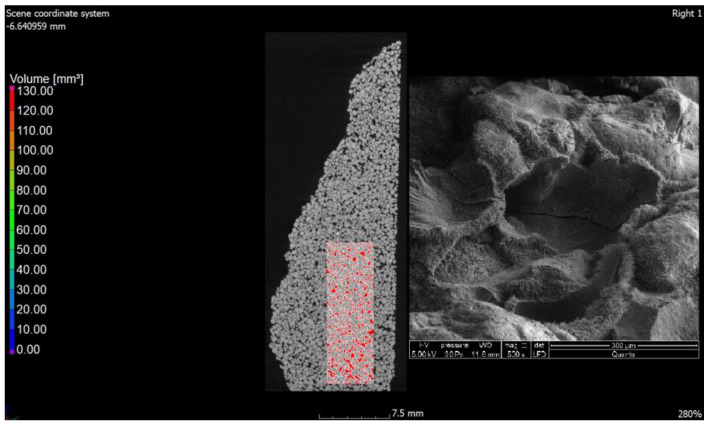
Cross-section through the reference material along with a photo from the scanning electron microscope; image 500×.

**Figure 9:**
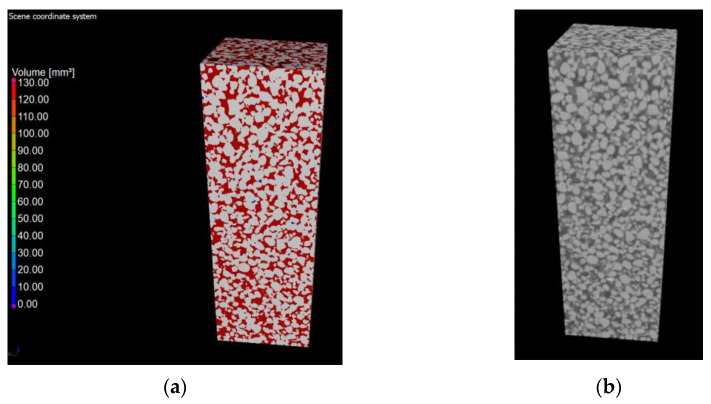
Cross-section through a sample of traditional sand-lime brick. (**a**) The pore content is marked in red;.(**b**) Cross-section through a sample of sand-lime brick with a visible arrangement of SiO_2_, CaO grains and free spaces of different transparencies, shapes and sizes.

The values of air content in the tested samples of the autoclaved products differ from each other by +/−1% (0.78% to be exact for these specific samples) and more air pores are present in the material modified with glass sand. The obtained difference may be due to the fact that the computed tomography method included a 3D analysis, and the obtained values were calculated on the basis of measuring the actual diameters of air voids in the tested element and the cross-section. The outer texture of the porous silicate material, on the other hand, was characterized by greater hardness and was less susceptible to mechanical damage. This difference may be associated with the fact that quartz sand is less reactive because it has a higher Mosh hardness (7) and can scratch glass [53]. The material strength was in the range of 10–20 MPa (the compressive strength test was the subject of the previous article [2,3]); therefore, it was similar to the reference brick, but the glass-modified material was more compact and uniform. The analysis of voids in the traditional silicate brick sample treated as the reference value showed that the pore content below 300 µm represented 0.02%, i.e., 0.06 mm^3^, while in the sample modified with recycled glass sand, it represented 0.8%, i.e., 2.5 mm^3^. The photos (Figure 6, Figure 7, Figure 8 and Figure 9) show images of a porous material, which is a traditional silicate brick. Figure 6 shows a fragment and a split of the reference brick, the distribution and size of the pores, which were taken with the tomograph together with a compilation of photographs from a scanning electron microscope (Figure 8) as a representation of phases that develop further in the pores of the porous material due to the hydration process (binding of the binder and water in the presence of sand and/or other components of the raw material, i.e., tobermorite). Figure 7 is a sample of sand-lime brick with a visible arrangement of SiO_2_, CaO grains and free spaces of different transparencies.

**Figure 10:**
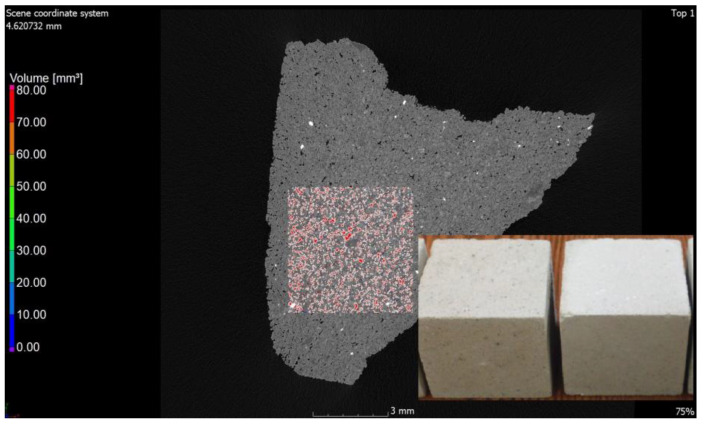
Photo of silicate brick modified with glass sand (GS) with an image of made bricks (dimensions 5 × 5 × 5 cm).

**Figure 11:**
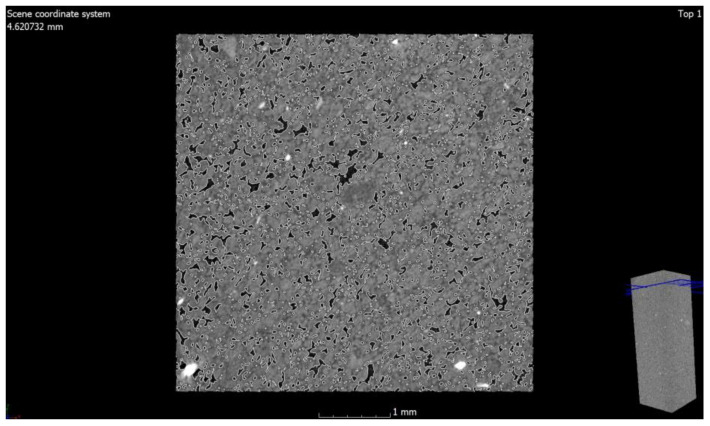
Cross-section through a sample of silicate material modified with glass sand. The drawings show a high number of small air voids.

**Figure 12:**
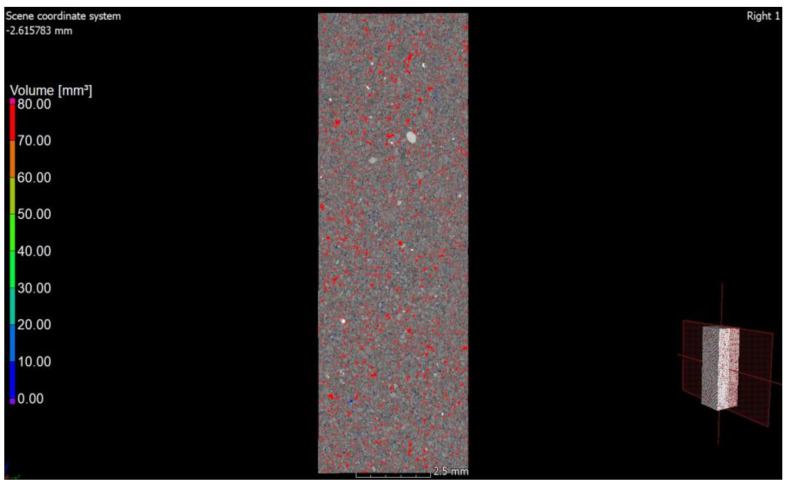
Cross-section of a sample of GS-modified silicate material.

The photo (Figure 8) shows both the pore arrangement in the traditional material and the image of its microstructure. The microstructure of silicate bricks, like concretes, consists of hydrated calcium silicates of various forms—from the amorphous C-S-H phase to crystalline phases (tobermorite, xonotlite, natrolite, etc.). A total of 90% of the silicate brick microstructure consists of crystalline phases, namely tobermorite phase, and these phases are formed in the pores. Amorphous phases, such as C-S-H, have larger specific surface areas, creating a system of smaller pores, while as the crystallization process progresses, the amount of voids generally increases because the crystalline phases have a smaller specific surface area. Recycled glass sand is also an amorphous substrate (similar to fly ash). Figure 10, Figure 11 and Figure 12 show the microstructure of a sample of silicate brick modified entirely with glass sand and made with dimensions of 5 × 5 × 5 cm (Figure 10) to emphasize the differences between the reference brick and the modified GS brick. The photo (Figure 10) shows both the texture of the silicate bricks with glass sand (GS) and the cross-section through the sample. The texture and structure of the material are more compact, albeit porous. The total number of pores is higher than in the reference sample; however, as shown in the image, the number of fine pores prevails.

**Figure 13:**
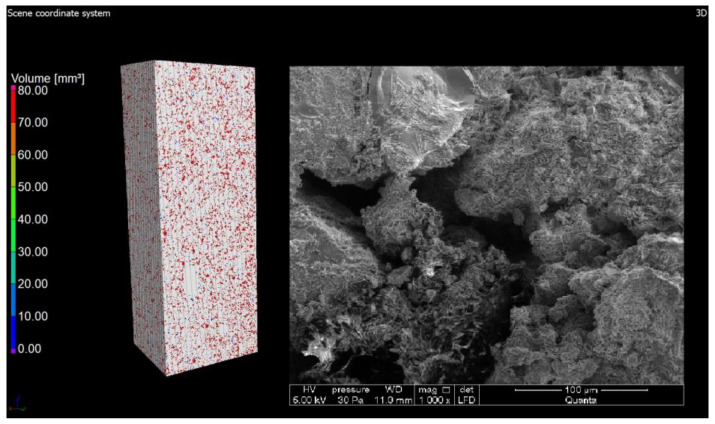
Image of the surface and microstructure of a material modified with glass sand; mag 1000×.

The photos (Figure 10, Figure 11, Figure 12 and Figure 13) show that pores with small diameters exhibit a greater frequency (as opposed to in the case of the reference brick). This indicates that glass sand is more reactive than quartz sand, which also affects the porous properties of the tested material. Figure 13 shows an image of the microstructure of the porous material (SEM), i.e., silicate brick, with the participation of GS and the change in the distribution and volume of the pores (Figure 13).

**Figure 14:**
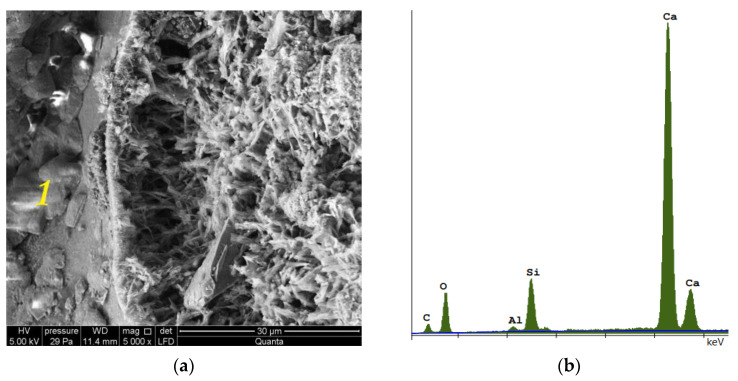
Photograph of the microstructure of traditional silicate brick (sand-lime) taken using SEM. (**a**) Picture of the microstructure of sand-lime sand brick. The image shows tobermorite in the form of thin plaques. (**b**) EDS spectrum of sand-lime sand brick in point 1.

**Figure 15:**
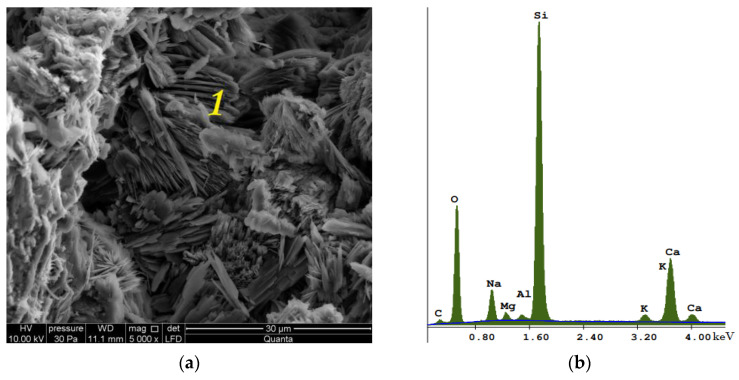
Photograph of the microstructure of silicate brick modified with glass sand and taken with the use of SEM; (**a**) gyrolite/natrolite—the figure shows systems in the form of flakes (1), thin sheets of ice that form into clusters, similar to gyrolite (1) or natrolite. (**b**) EDS spectrum glass sand modified silicate brick in point 1, visible Na as a component of glass sand and an element responsible for the crystallization toward gyrolite or natrolite.

Photo 14 shows an image of the microstructure of a reference (traditional, Figure 14a) brick, together with the EDS spectrum (Figure 14b). Photo 15 shows a silicate brick, which was made by completely replacing quartz sand by glass sand (EDS spectrum, Figure 15b). The C-S-H phase crystallized and transformed into natrolite and gyrolite within 5 years of production (Figure 15a). The sample modified by glass sand, both in the CT and SEM photos (Figure 13), shows a more uniform internal structure and texture (with a compressive strength of 20 MPa). This may be related to the hydration process, which for both materials takes place in a different way, i.e., the hydration temperature of the lime binder with water in the presence of quartz sand for the traditional (reference) material reaches the value of 70–80 °C, while the hydration temperature of the lime binder with water in the presence of glass sand (90% GS, 0% QS) reaches a maximum of 42 °C. This value increases with a proportional increase in the quartz sand content in the modified material. Recycled glass sand (GS) also has a higher moisture content, which can define the hydration process. Moreover, the silicate mass with the participation of glass sand has the properties of clay and is also more plastic in comparison to the silicate mass with the participation of quartz sand (QS).

**Figure 16:**
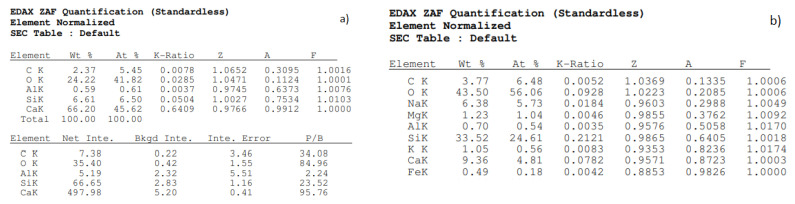
Elementary composition of silicate brick (SEM); (**a**) traditional (reference sand-lime brick 90% QS); (**b**) glass sand modified by 90%.

The images on Figure 15 show changes, especially in the shape of the pores and their span (volume), and next to tobermorite the C-S-H (amorphous) phase crystallized and resulted in the formation of natrolite and gyrolite (clusters of small flakes). Figure 16 shows the elemental composition of traditional silicate brick (Figure 16a) and the one modified with glass sand (Figure 16b). An aim of this article was to illustrate how the pores are arranged in traditional (reference) sand-lime brick (QS), as well as in bricks modified with glass sand (GS).

### 3.2. Mercury Porosimetry

**Figure 17:**
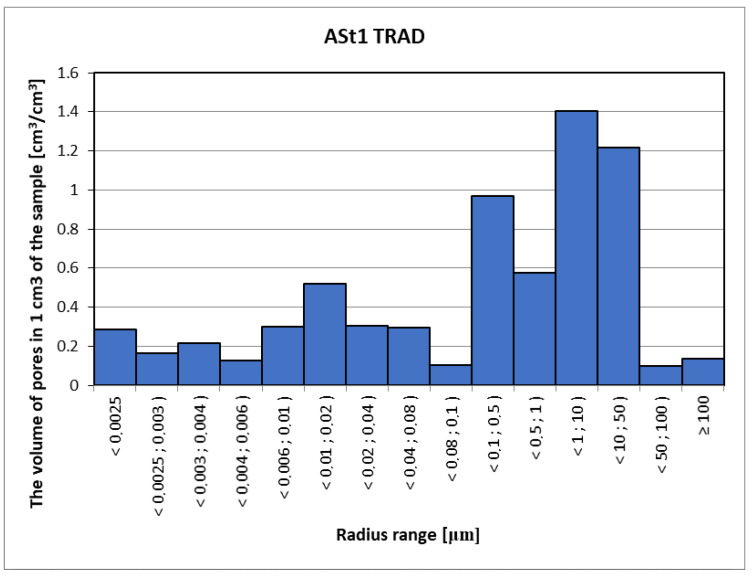
Distribution of the volume and range of pore radii for traditional sand-lime brick.

**Figure 18:**
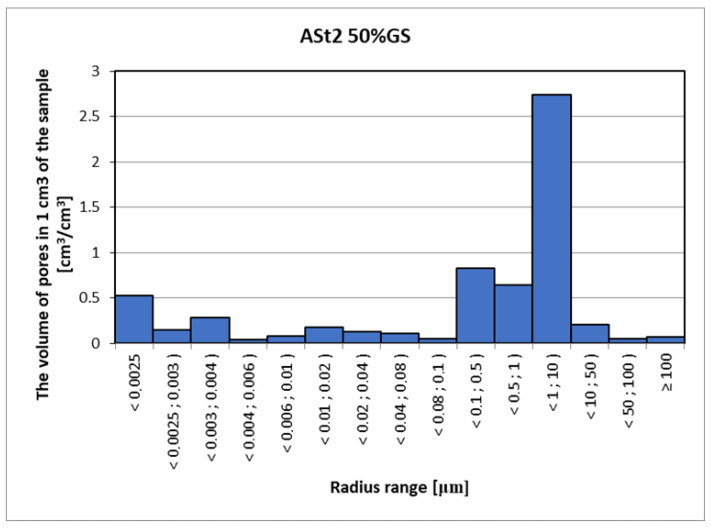
Distribution of the volume and range of pore radii for 50% GS modified silicate brick (i.e., 50% QS + 50% GS in relation to the share of sand (90%) in the total silicate mass, the remaining 10% being 7% CaO and 3% H_2_O).

**Figure 19:**
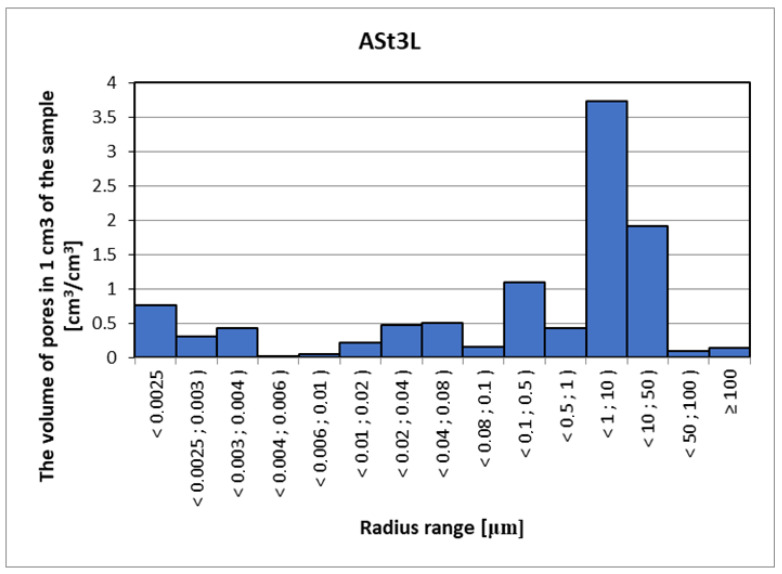
Distribution of the volume and the range of pore radii for silicate brick modified with 90% glass sand (GS).

Mercury porosimetry is a method often used to measure the pore volume in a material, but unlike computed tomography, it is a destructive method, wherein there is a risk of damaging the structure of the porous skeleton as a result of forcing high-pressure mercury into the material. With this method, special care should also be taken due to the harmfulness of mercury. The analysis of the porosimetric test results (Figure 17, Figure 18 and Figure 19) considers the fact that mercury is forced into pores with a diameter of 0.01 µm and smaller using very high pressure (over 100 MPa). Pores in the material are a network connected by narrow passages, and the material sample and the target material itself are not able to withstand such a load, and therefore some of the pores are destroyed due to the strong influence of mercury. At this point, however, the possibility of injecting mercury into the bottle pores should be considered without drastically destroying the structure of the material. The maximum pressure of 420 MPa is necessary to force mercury into pores with a diameter of 0.006 µm [3]. Assuming that the destructive pressure is close to the tensile strength of the tested material, with mercury penetration into the pores with radius dimensions ≤ 0.02 µm, the damage process will begin, changing the image of the actual pore distribution in the tested material. The density of the samples was as follows: ASt1 TRAD: 2.62 kg/dm^3^, ASt2 50% GS: 2.33 kg/dm^3^ and ASt3L: 2.21 kg/dm^3^.

**Figure 20:**
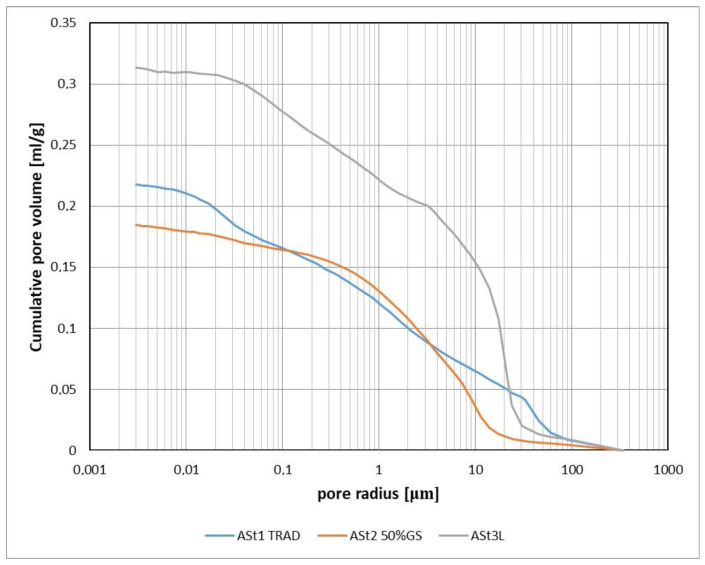
The cumulative volume of pores in the samples of silicate material: traditional (blue color), modified with 50% glass sand and 50% quartz sand (red color) and the material fully modified (90%) with glass sand (gray color).

The distributions of the Ast1 Trad and Ast2 50% GS curves (Figure 20) are similar, which indicates that the uniform proportion of aggregate in the materials modified with glass sand makes for properties similar to those of the traditional sand-lime bricks. The third curve (gray) is a graph of a material in which the crystalline quartz sand has been completely replaced by recycled glass sand with an amorphous structure, which is more susceptible to reactions and changes in the autoclaving process. This curve shows that pores with smaller diameters have a greater share in the material, and the share of pores with diameters above 1 µm decreases.

## 4. Discussion

The degree of content of individual phases in the structure of materials and the method of obtaining or processing substrates for modification, and then the production of materials itself, determine the final properties of each material. It seems important to accurately determine the porous structure of building materials and the method for this purpose, e.g., due to the possibility of damaging the structure of the micropores during the tests, which should always be taken into account. Numerous modifications cause changes in the structure of known materials, and these may have a positive, negative or neutral effect on the porosity and changes in pore volume over time (under environmental conditions). The compounds and impurities found in both quartz sand and glass sand are important during the synthesis and crystallization of the phases. For example, aluminum influences the formation and synthesis of the tobermorite phase, thus reducing the specific surface area. The glass cullet, in turn, causes the crystallization of the C-S-H phase toward natrolite and gyrolite. It is now important to examine the amount of crystalline and amorphous phases in the tested bricks (percentage or weight) and the quality of these phases. The next step of this research will consist in determining the changes in the microstructure of the material modified with glass sand after 5 and 7 years from the date of their production in 2015. A geochemical equilibrium-based simulation will be performed using the GEMS-PSI program, and the samples will be re-examined using a computer tomograph (after another 12 and 24 months). The pore space of the autoclaved materials (traditional and modified with glass sand) will also be examined by the Archimedes method.

## 5. Conclusions

The materials covered by this study were silicate bricks with a traditional composition and silicate bricks modified by glass sand in the amounts of 0% (reference material made only on the basis of quartz sand), 50% quartz sand, 50% recycled glass sand and 90% recycled glass sand (total elimination of quartz sand).

The important task of this article was to present a non-destructive method in the form of computed tomography to visualize porous materials, such as silicate bricks.

Using computed tomography, two types of bricks were examined: traditional (reference) sand-lime bricks, the main component of which was quartz sand (90%), and bricks entirely modified with glass sand from recycled glass (90% GS). The experiment was to show how the pores are arranged in the traditional (reference) sand-lime brick as well as in the brick modified with glass sand, as well as the difference in the microstructure of both materials. The computed tomography examination was compared with the mercury intrusion porosimetry examination. The porosimetry method is widely used to analyze porous materials, but studies have shown that this method can damage the internal skeleton of the material by violently and forcefully acting on the sample with mercury under pressure. The analysis of the voids in the traditional silicate brick sample treated as the reference value showed that a pore content below 300 µm represented 0.02%, i.e., 0.06 mm^3^, while in the sample modified with recycled glass sand, it represented 0.8%, i.e., 2.5 mm^3^. Both methods can be compared with each other and give relatively reliable results; however, non-destructive computed tomography can be performed many times without disturbing the structure and skeleton of the tested material.

## Abbreviations

QS	Quartz sand
GS	Glass sand
U	Heat transfer coefficient (W/(m^2^·K))
CT	Computed tomography
SEM	Scanning electron microscope
ASt1 TRAD	Traditional (reference) silicate brick (lime-sand, 90% quartz sand)
ASt2 50%GS	Silicate brick with 50% glass sand and 50% quartz sand in relation to the sand volume in the raw material mass (90% SiO_2_)
AST3L	90% glass sand (complete elimination of quartz sand)
